# Hall Sensors for Extreme Temperatures

**DOI:** 10.3390/s110100876

**Published:** 2011-01-14

**Authors:** Jakub Jankowski, Semir El-Ahmar, Maciej Oszwaldowski

**Affiliations:** Institute of Physics, Poznan University of Technology, Nieszawska 13a, 61-965 Poznan, Poland; E-Mails: jakub.jankowski@doctorate.put.poznan.pl (J.J.); semir.el-ahmar@put.poznan.pl (S.E.-A.)

**Keywords:** Hall sensors, n-InSb/GaAs, extreme temperatures, magnetic sensors

## Abstract

We report on the preparation of the first complete extreme temperature Hall sensor. This means that the extreme-temperature magnetic sensitive semiconductor structure is built-in an extreme-temperature package especially designed for that purpose. The working temperature range of the sensor extends from −270 °C to +300 °C. The extreme-temperature Hall-sensor active element is a heavily n-doped InSb layer epitaxially grown on GaAs. The magnetic sensitivity of the sensor is *ca.* 100 mV/T and its temperature coefficient is less than 0.04 %/K. This sensor may find applications in the car, aircraft, spacecraft, military and oil and gas industries.

## 1. Introduction

Electronic devices are constantly entering into new fields of application that frequently expose them to harsh environments, particularly to temperatures that are far beyond the common “room temperature” range. These “extreme temperatures” (ET) encompass both the lowest (cryogenic) temperatures, close to absolute zero (−273.16 °C), and very high temperatures, 300 °C, or even above. Demands for the applications of various devices in such extreme temperatures come mainly from the car, aircraft, spacecraft, military and oil and gas industries.

Among the ET electronic devices which are sought after, are magnetic field sensors based on the Hall effect. By ET electronic devices one understands devices that can be used both in the lowest and in the highest temperatures *i.e.*, in the temperature region that extends from about 1 K to about 600 K. Obviously, in the case of semiconductor devices, it is usually much more difficult to achieve a good performance at high temperatures. Hence, the candidates for an extreme temperature Hall sensor (ETHS) should be recruited from the existing or potential high temperature Hall sensors (HTHS).

It is commonly recognized [1–5] that the high temperature electronics should be based on wide band-gap materials. This is because of the thermal stability of their electrical parameters at elevated temperatures. The weak point of these materials is, however, a low electron mobility, which results in a lower magnetic sensitivity of the sensors made of them in comparison with the narrow band-gap materials.

Among the wide band-gap semiconductors, SiC [6] and AlGaN [7] were examined as potential candidates for the preparation of an HTHS. SiC is indeed interesting for the preparation of an HTHS, but not for an ETHS, as it has a very small electrical conductivity below 200 K.

The situation is better with GaN. It happens that an unintentionally doped AlGaN/GaN heterojunction has the unique property of forming a two-dimensional electron gas (2DEG) at the interface. The electron density of the 2DEG depends very weakly on temperature up to 300 °C [7,8], which means that up to this temperature stable 2DEG can be a basis for the preparation of HTHS. For this purpose both simple AlGaN/GaN heterojunctions [7,9,10] and field-effect transistor structures based on this heterojunction [11–13] were examined. The electron mobility of the 2DEG in the heterojunction is about 1,000 cm^2^/(Vs), and strongly decreases with an increase in temperature to a value of about 300 cm^2^/(Vs) at 300 °C [8,13]. This small mobility value and its strong temperature dependence are disadvantages from the point of view of the application in Hall sensors. On the contrary, above room temperature the temperature coefficient of the Hall voltage generated at the heterojunction can be as low as −7 × 10^−4^ %/°C [12], which is the best result known for semiconductor materials.

Here we show that heavily donor doped InSb thin films are an excellent material for the preparation of an ETHS working in the temperature range from 2 K (−273 °C) to 573 K (+300 °C). In previous papers we have shown that Hall sensor structures made of such thin films are excellent materials for the preparation of HS designed for working both in the low temperature range (2–250 K) [14] and in the high temperature range (273–573 K) [15]. Therefore, the main tasks of the present work were the optimization of the sensor structure parameters and the elaboration of the sensor package for the temperature range 2–573 K. In the case of the HT electronics, the HT package is a serious problem that has to be separately solved for a given device type [1–5]. Here we give the description of the parameters and characteristics of an ETHS equipped in an ET package, and not the ETHS sensitive structure only.

## 2. Sensor Preparation

The Hall sensor structure preparation method has been described in detail in previous papers [14,15]. Hence, here we give only a brief description of the method. The sensitive element of the sensors is an *n*-InSb thin film, about 1 μm thick, epitaxially grown on a GaAs (100) substrate by the flash-evaporation epitaxy method. During the film growth, donor doping with tin is performed. As a result, the electron concentration in the film is (2–3) × 10^18^ cm^−3^. This concentration is optimum for preparing a HS with a very good thermal stability of the main parameters over a wide range of temperatures. The thickness of 1 μm is also chosen to optimize the sensor parameters.

The electron mobility in InSb thin films decreases with the film thickness below 3 μm, and particularly strongly below 1 μm [16]. Hence, the advantage from the Hall voltage increase resulting from the film thickness decrease is largely reduced or lost because of the increase in sensor resistance. Moreover, thinner InSb films, about 1 μm in thickness, have a very weakly temperature dependent resistance [14] in a broad temperature range, which allows biasing the 1 μm HS also in the constant voltage regime [17].

**Figure 1.**
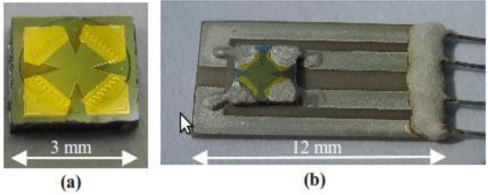
Extreme-temperature Hall sensor; **(a)** ETHS structure. Maltese cross-shaped InSb layer is plated with Cr-Au electrodes, and **(b)** ETHS in an open ET package.

In order to protect the sensitive InSb thin-film structure from the hot ambient, it is covered with a protective layer. For that purpose, a 0.1 μm SiO*_x_* (*x* ≤ 2) layer deposited in vacuum is used. After the InSb films deposition, the films are photolitographically cross-shaped and equipped with 0.5 μm Cr/Au electrodes by vacuum evaporation. Finally, the GaAs chip is divided onto 3 × 3 mm^2^ sensor structures. The resulting HS structure is shown in Figure 1(a).

The HS structure is mounted in a HT package, as shown in Figure 1(b). The package is based on a 12 × 6-mm^2^ base plate made of Al_2_O_3_ or AlN. The base plate has four electric traces formed by silk-screen printing of thick AgPt films. The Hall structure is attached to the base plate with HT Al_2_O_3_ paste (PELCO High Performance Ceramic Adhesive, www.tedpella.com). In the next step the electric connections to the conducting paths are formed. The contacts to the gold electrodes of the InSb film are made by a standard thermocompression bonding with a 100-μm diameter gold wire. The gold wire and the electrode are then covered with HT silver paste (AMEPOX, Electon 40AC paste, www.amepox-mc.com), therefore, the connection is formed of HT silver paste with a gold core. Such a contact has very good mechanical and electric properties in a wide temperature range. On the other end of the base plate, the contacts between the external wires and the four electric traces are formed by HT silver paste covered with HT Al_2_O_3_ paste for mechanical strengthening.

The package was tested in the high temperature range by thermal cycling of a group of the Hall sensors between room temperature and 300 °C and prolonged annealing at 300 °C [18]. All the sensors passed the test positively. The tests of HS structures in the low-temperature range have been described previously [14]. For the present purpose, we repeated the tests on the HS structures mounted in the HT package. These tests showed that the HT package [18] can also be used at the low temperatures. Thus, the package can be used in the full temperature range allowed for the ETHS. At the present stage of the technology development, no attempt has been made either for the miniaturization of the HS structure [Figure 1(a)] or for the package [Figure 1(b)]. The package shown in the figure is an open version. For mechanical protection, the package can be equipped with a lid.

It was established that the present technology yields the Hall sensors having the following parameters:

**Table:** 

Working temperature range, Δ*T*	−270 °C to +300 °C
Working magnetic field range, Δ*B*	0–5 T
Input/output resistance, *R*	≈ 10 Ω
Nominal driving current, *I*_n_	50 mA
Maximum driving current, *I*_max_	100 mA
Magnetic field sensitivity, *S*	≈ 100 mV/T
Temperature coefficient of resistance, |*α*|	<0.10 %/°C
Temperature coefficient of magnetic sensitivity, |*β|*	<0.04 %/°C

The absolute values of the temperature coefficients are given as average values in the full temperature range between −270 °C and +300 °C. For temperatures below and above room temperature, the coefficients *α* and *β* are slightly lower and higher, respectively.

## 3. Stabilization of Sensor Parameters at High Temperatures

Electrical and mechanical properties of electronic devices can be modified by annealing of the working semiconductor structure and the package during high-temperature measurements. The heat treatment can change both the properties of the working semiconductor structure and the package. The effect of annealing is particularly likely to occur in thin-film semiconductor structures because the thin films are, as a rule, obtained at conditions different from thermodynamic equilibrium. Processes such as atomic diffusion, surface migration and chemical reactions between different parts of the system and the environment and defect generation are expected to occur during the annealing. In the case of InSb, the abovementioned processes are particularly likely to occur at the maximum allowed temperature of 300 °C because this temperature is rather close to the melting point of InSb (525 °C).

**Figure 2.**
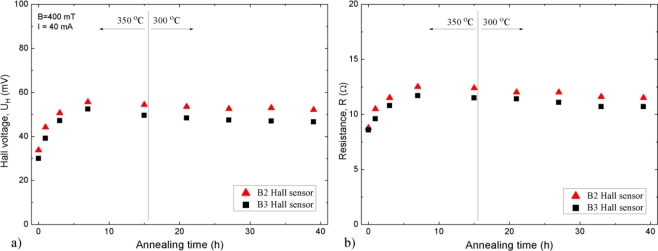
Effect of annealing on ET Hall sensors B2 and B3 performed at temperatures 350 °C and 300 °C; **(a)** change of Hall voltage, *U*_H_, **(b)** change of input resistance, *R*.

In view of the above, we considered it necessary to perform an investigation of the effect of prolonged annealing on the electric properties of the sensors. The annealing procedure was divided into two parts, as shown in Figure 2, where typical results of measurements representing eight investigated Hall sensors are displayed. The data are presented for sensors labeled B2 and B3 taken as examples.

In the first part, the annealing was performed at 350 °C in air. This annealing lasted 15 h with breaks for performing measurements. This period appeared to be sufficient to roughly stabilize the sensor Hall voltage, *U*_H_, and the resistance, *R*, as may be concluded from Figure 2. The temperature of 350 °C, which is above the allowed 300 °C*,* was applied to accelerate sample stabilization [15]. In the second part, further annealing in air, lasting up to a total of 39 h, was performed at the maximum allowed sensor temperature of 300 °C. As may be seen in Figure 2, in this second part changes in the parameters are small and after 30 h they are practically negligible.

After the stabilization through the prolonged annealing process, the sensor parameters do not change in the course of further thermal cycling up to 300 °C. Thus, the sensors assure reliable accurate magnetic field measurements.

## 4. Sensor Characteristics

**Figure 3.**
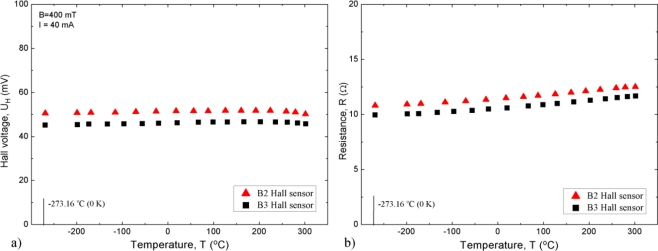
Temperature dependence of **(a)** Hall voltage *U*_H_, and **(b)** resistance *R* sensors B2 and B3.

The basic sensor characteristics are again presented for sensors B2 and B3. The temperature dependences of the sensor Hall voltage, *U*_H_, and resistance, *R*, in the temperature range between −269 °C and +300 °C (4.2–573 K) are shown in Figure 3.

The Hall-voltage temperature dependence is small over the whole temperature range. This dependence results in a very small temperature coefficient |*β*| < 0.04 %/K. Actually, we only observed measurable temperature changes above room temperature. These changes are even smaller in the Hall sensors having magnetic sensitivity lower by about 50% than that of sensors B2 and B3. Hall sensors made of InSb layers having electron concentration higher by 50%, have such low sensitivities. Thus, the coefficient *β* can be improved at the expense of the sensitivity.

The temperature dependence of the resistance is also small and results in an average temperature coefficient |*α*| < 0.10 %/°C. This coefficient, with a small absolute value, can be negative as well as positive, depending on the preparation technology of the InSb layers [17]. In particular, it can be made by technological means exceptionally small, as small as 0.01 %/°C. In such a case, the Hall sensor is suitable for biasing in the constant voltage regime [17].

**Figure 4.**
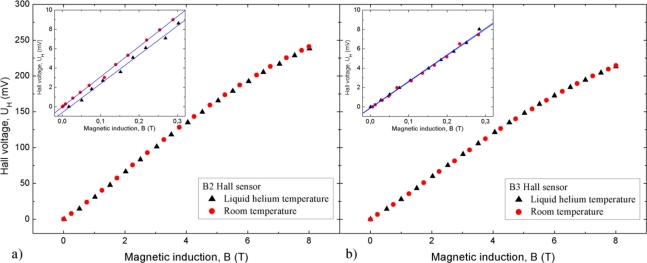
Field dependence of Hall voltage *U*_H_ in range (0–8) T at 20 °C and −269 °C for sensors B2 and B3. This d0ependence at low magnetic fields is given in insert.

The dependence of *U*_H_ of the sensors on magnetic field *B* at room temperature and −269 °C is shown in Figure 4. The dependence *U*_H_(*B*) is linear between 0 T and 5 T and it decreases at higher magnetic fields. The dependence for the field smaller than 0.3 T is shown in the insert. As may be seen, it is linear. However, it should be mentioned that the dependence *U*_H_(*B*) is linear between 0 T and 10 T in the case of the sensors having the magnetic sensitivity smaller by about 50% than that of sensors B2 and B3.

**Figure 5.**
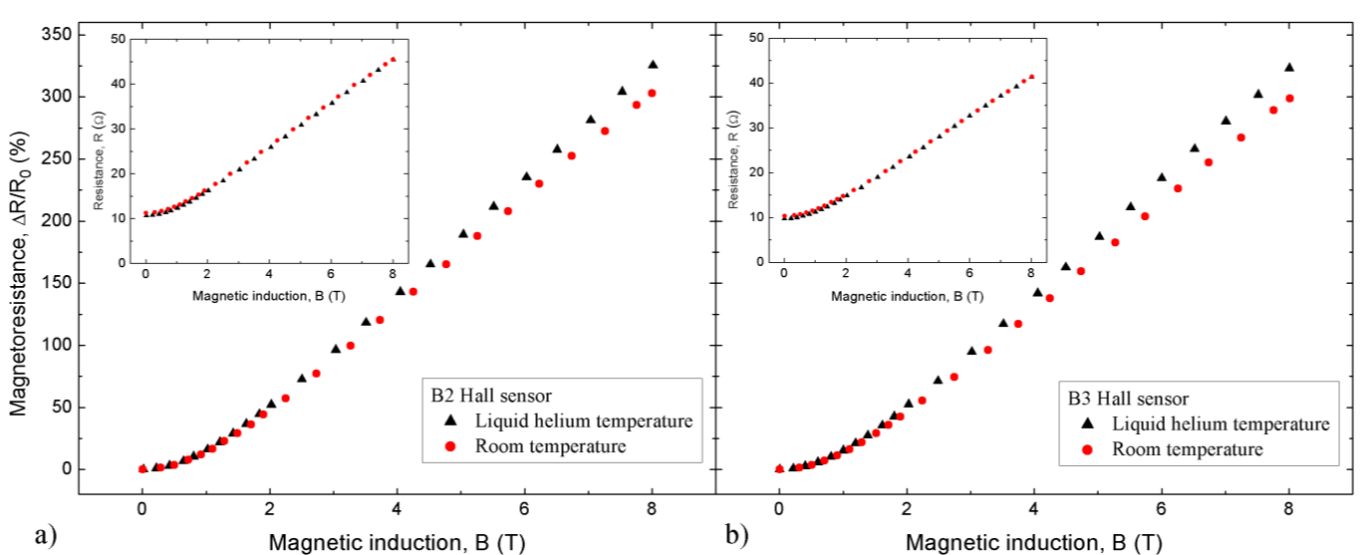
Field dependence of relative magnetoresistance Δ*R*/*R*_0_ in the range (0–8) T at 20 °C and −269 °C for sensors B2 and B3. This dependence was obtained from the measured dependence *R*(*B*) shown in inserts.

The dependence of the sensors relative magnetoresistance, Δ*R*/*R*_0_, on magnetic field *B* at room temperature and −269 °C is shown in Figure 5. The relative magnetoresistance Δ*R*/*R*_0_, was calculated from the relation:

(1) $$\Delta R/R_{0}\equiv [R(B)-R(0)]/R(0)$$

where *R*(*B*) and *R*(0) are the values of resistance at B = B and B = 0 respectively. The *R*(*B*) dependence is given in the insert. It may be seen in the figure that the magnetoresistance is slightly higher at low temperatures in comparison to that at room temperature. The magnetoresistance is quadratic in magnetic fields below about 0.3 T, and is roughly linear at stronger magnetic fields.

## 4. Summary and Conclusions

This paper presents the description of the first completely developed extreme-temperature Hall sensor (ETHS) in which a sensitive ETHS semiconductor structure is mounted into an ET package. The sensitive structure is made of heavily donor-doped InSb thin films epitaxially grown on semi-insulating GaAs substrates. The structure is chemically and mechanically protected with a SiO_2_ layer. After appropriate annealing procedure of such a protected structure, it becomes thermally stable and resistant against the action of active environmental gases, such as oxygen, at elevated temperatures. The HT package consists of a substrate, which can be either Al_2_O_3_ plate or AlN plate. The latter has very high thermal conductivity (140–170 W/m·°C) because only diamond has a higher conductivity. As may be seen from Figure 3(a), the Hall voltage depends very weakly on temperature over the whole temperature range. The resulting temperature coefficient of the magnetic field sensitivity may be both positive as well as negative, but its absolute value |*β*| < 0.04 %/°C. Both the sign and the value of *β* depend on the details of the InSb layer preparation. As observed from Figure 3(b), the temperature dependence of resistance is also relatively small and results in a temperature coefficient |*α*| < 0.10 %/°C. As explained in Ref. [17] this coefficient can also be modified by technological means.

The magnetic field dependence of the Hall voltage is linear for *B* < 0.3 T (insert in Figure 4). This linearity continues up to 1 T. Above 5 T, the *U*_H_(*B*) dependence tends to reach saturation. In this magnetic field range, one can afford to use the sensor with minimal magnetic field sensitivity. Samples with a sensitivity of about 50% lower than that of sensors B2 and B3 have such a minimal sensitivity. They show a linear dependence of U_H_(*B*) up to *B* ≥ 10 T.

The sensors show relatively large magnetoresistance (Figure 5). This is because of the high electron mobility of the InSb layers. Above 2 T, the magnetoresistance is roughly linear in *B*. This means that at the high magnetic fields, the HS can be used as a magnetoresistor, a two-terminal device (HS is a four-terminal device).

The resistance of the sensors is about 10 Ω. This is a small value, allowing a relatively large driving current of 50 mA. Thus, the power dissipated in the sensor structure is about 25 mW. This power may, however, be too high at very low temperatures because the power dissipated can warm the system. Thus, it may be necessary to decrease the driving current. According to our measurements, the values of the magnetic sensitivity and the resistance are independent of the current value in the range 0.1–10 mA both at the liquid-helium and room temperatures. The allowed driving current at very low temperatures depends on the measured system volume and thermal conductivity, and therefore cannot easily be predicted.

An important positive feature of the elaborated ETHS is it is electrically and mechanically robust. The low resistance and the high driving current allow using them in high electric noise environments. The sensors were also tested regarding resistance to the neutron irradiation [19]. They showed a good irradiation resistance up to neutron fluence of 10^17^ neutrons/cm^2^. The scientific extension of the presented paper compared to the previous ones [14,15] is following:

1. Application of a new casing that enables using the Hall sensor in the wide range of temperatures, from −270 °C to +300 °C. In this way the first complete ETHS has been manufactured.
2. Changing the InSb layer technology that enabled decreasing the driving current and the temperature coefficient of the resistance change. This is an important development of the sensor for low temperature measurements of magnetic field.
3. Development of a procedure of the sensor parameters stabilization by prolonged annealing in air.

Since we present the first fully-developed ET Hall sensor, it is impossible to compare its properties with other sensors of this sort. Unfortunately, even comparisons of the properties of our InSb/GaAs structure with the properties of the other candidate semiconductor structures can be performed only to a limited extent because of lack of data. As mentioned in the Introduction, the most promising candidate structure is that of the AlGaN/GaN heterojunction. Alas, neither data on the magnetic field dependence of Hall voltage and resistance nor optimum value of the driving current has been published so far for that Hall structure. In view of this, we can only conclude that, at high temperatures, the temperature coefficient of the magnetic sensitivity of our InSb/GaAs structure is low. However, the input/output resistance of the InSb/GaAs structure is relatively low and its temperature coefficient of this structure resistance is also low. General properties of the HSs based on heavily donor-doped InSb/GaAs heterostructures and comparison of their basic parameters with those of commercially available HSs have been described in a previous paper [20].
